# The complete mitogenome of the Greytail angelfish *Chaetodontoplus poliourus* and its phylogenetic relationships (Perciformes: Pomacanthidae)

**DOI:** 10.1080/23802359.2021.1933632

**Published:** 2021-11-03

**Authors:** Kang-Ning Shen, Chih-Wei Chang

**Affiliations:** a Aquatic Technology Laboratories, Agricultural Technology Research Institute, Hsinchu, Taiwan, R.O.C.;; b Marine Ecology and Conservation Research Center, National Academy of Marine Research, Kaohsiung, Taiwan, R.O.C.;; c Department of Oceanography, National Sun Yat-sen University, Kaohsiung, Taiwan, R.O.C.;; d National Museum of Marine Biology and Aquarium, Pingtung, Taiwan, R.O.C.

**Keywords:** *Chaetodontoplus poliourus*, Pomacanthidae, next-generation sequencing, mitogenome

## Abstract

The mitogenome of the Greytail angelfish *Chaetodontoplus poliourus* (Pomacanthidae) was decoded using next-generation sequencing techniques. The *de novo* assembled mitogenome consists of 16,961 bp, including 13 protein-coding genes, 2 ribosomal RNAs and 22 transfer RNA genes. The gene arrangement is identical to the other available Pomacanthidae mitogenomes submitted to NCBI. The overall base composition of the *C. poliourus* mitogenome was estimated to be 28.0% A, 30.9% C, 15.8% G and 25.3% T. The phylogenetic analysis of the *C. poliourus* mitogenome suggests a closer genetic relationship with Vermiculated angelfish *Chaetodontoplus mesoleucus* as expected from their similar color patterns. The overall pairwise identity except D-loop is 93.7% for these two sister species. The decoding of the *C. poliourus* mitogenome has enriched gene database for further evolutionary studies and conservation assessments of this uncommon and newly described marine angelfish.

The Greytail angelfish, *Chaetodontoplus poliourus* Randall and Rocha, 2009 is one of the newest described species of marine angelfish (Pomacanthidae). It was previously regarded as a color variant of Vermiculated angelfish *Chaetodontoplus mesoleucus* (Bloch, 1787) because it looks very similar in color patterns at first glance. The main distinction between the two species is a bright yellow tail in the Vermiculated angelfish and a clear tail in the Greytail angelfish. Despite the similarity in coloration, these two species have different distribution ranges and only overlapped in Eastern and Southern Indonesia. They were sometimes found in sympatry on the same reef but without interaction (Debelius et al. 2003). This species has been recorded from Bali, Flores (Southern Indonesia) to Papua New Guinea, the Solomon Islands and Palau (Randall and Rocha 2009). It occurs at depths of 2–25 m, inhabits coral-rich areas in back-slopes and lagoons. Although *C. poliourus* is at Least Concern in IUCN Red List (Pyle et al. 2010), conservation status is still unclear due to the rarity of observations compared to *C. mesoleucus*. Further information on its population genetic structure is needed for the potential risk assessment of extinction.

Genomic DNA of *C. poliourus* was extracted from muscle tissue of a specimen collected from Bali, Indonesia (imported for the aquarium fish trade) on 11 May 2012 and vouchered in Agriculture Technology Research Institute (voucher no: ATRI333). The TruSeq DNA Library Preparation Kits were used to prepare DNA libraries with insert sizes from 500 to 700 bp for Illumina Hiseq2500 sequencer. The next-generation sequencing reads of *C. poliourus* were *de novo* assembled by Geneious V9 software (Auckland, New Zealand) to produce a single, circular and complete mitogenome with an average coverage of 82.2 × (5594 out of 11,671,004 reads). Protein-coding genes, ribosomal RNA genes and transfer RNA genes were identified using MITOS (Bernt et al. 2013). Raw reads were deposited in a short read archive database in NCBI (SRA accession no. SRR13276947-8). Multiple alignments (MAFFT, Katoh et al. 2019) with entire genomes and phylogenetic analyses using the Maximum-likelihood method (Harris and Stocker 1998) under the best fitted model GTR + G + I mutation model with 1000 bootstrap tests were performed with MEGA7.0 (Kumar et al. 2016). Partial deletion was choice as the method to treat missing data and alignment gaps for phylogenetic tree construction in MEGA7.0. The Pomacanthidae species from different genera were chosen for phylogenetic analysis, including *Apolemichthys griffisi* (NC027592), *Centropyge heraldi* (NC027968), *Chaetodontoplus conspicillatus* (KP033452), *Chaetodontoplus mesoleucus* (KP218262), *Chaetodontoplus septentrionalis* (AP006007), *Genicanthus lamarck* (NC027972), *Holacanthus tricolor* (NC027586), *Paracentropyge multifasciata* (NC027599), *Pomacanthus imperator* (NC026304) and *Pygoplites diacanthus* (KP898265) with *Chaetodon auripes* (NC009870) used as outgroup. The evolutionary distances were computed using the *p*-distance method (Nei and Kumar 2000). Geneious V9 software was employed to draw the mitogenome map of *C. poliourus* (NCBI Accession Number MH517023) which is 16,961 bp in size and contains 28.0% A, 30.9% C, 15.8% G and 25.3% T.

The *mt-nd1*, *mt-nd2*, *mt-co1*, *mt-co2*, *mt-atp8*, *mt-atp6*, *mt-co3*, *mt-nd3*, *mt-nd4l*, *mt-nd4*, *mt-nd5*, *mt-cyb* genes, two ribosomal RNA genes as well as 14 tRNAs were found on the H-strand while the *mt-nd6* as well as other eight tRNAs were found on the L-strand. The gene arrangement is identical to the other available *Chaetodontoplus* mitogenomes that were submitted to NCBI. The *mt-nd1* gene started with the codon ATC; *mt-co1* gene started with the codon GTG; *mt-nd5* started with ATT and all other protein-coding genes started with ATG (*mt-nd2*, *mt-co2*, *mt-atp8*, *mt-atp6*, *mt-co3*, *mt-nd3*, *mt-nd4l*, *mt-nd4*, *mt-nd6*, *mt-cyb*). *mt-co2* and *mt-nd4* genes terminated with the codon AGA, *mt-nd3* and *mt-nd5* genes terminated with TAG, and most genes terminated with TAA. The maximum likelihood phylogenetic analysis suggested a earliest diverging lineage of genus *Chaetodontoplus* in Pomacanthidae (Figure 1), followed by *Pomacanthus* and other genera were clustered together while Bellwood et al. (2004) suggest *Pomacanthus* as earliest diverging lineage and Gaither et al (2014) and Frédérich et al (2017) data placed *Pomacanthus* and *Chaetodontoplus* as sister lineages. The phylogenetic relationships of *Chaetodontoplus* spp. have not been comprehensively surveyed, only four out of 13 species have been mitogenome decoded (including *C. poliourus*). The phylogenetic tree from this study suggests a closer genetic relationship between *C. poliourus* and *C. mesoleucus* with a *p*-distance of 6.3%. While 7.1% was found between *C. conspicillatus* and *C. septentrionalis*, 13.5% between *C. conspicillatus* and *C. poliourus*/*C. mesoleucus*, 13.8% between *C. septentrionalis* and *C. poliourus/C. mesoleucus*. It Suggests that *C. poliourus* is a valid and sister species of *C. mesoleucus*.

**Figure 1.**
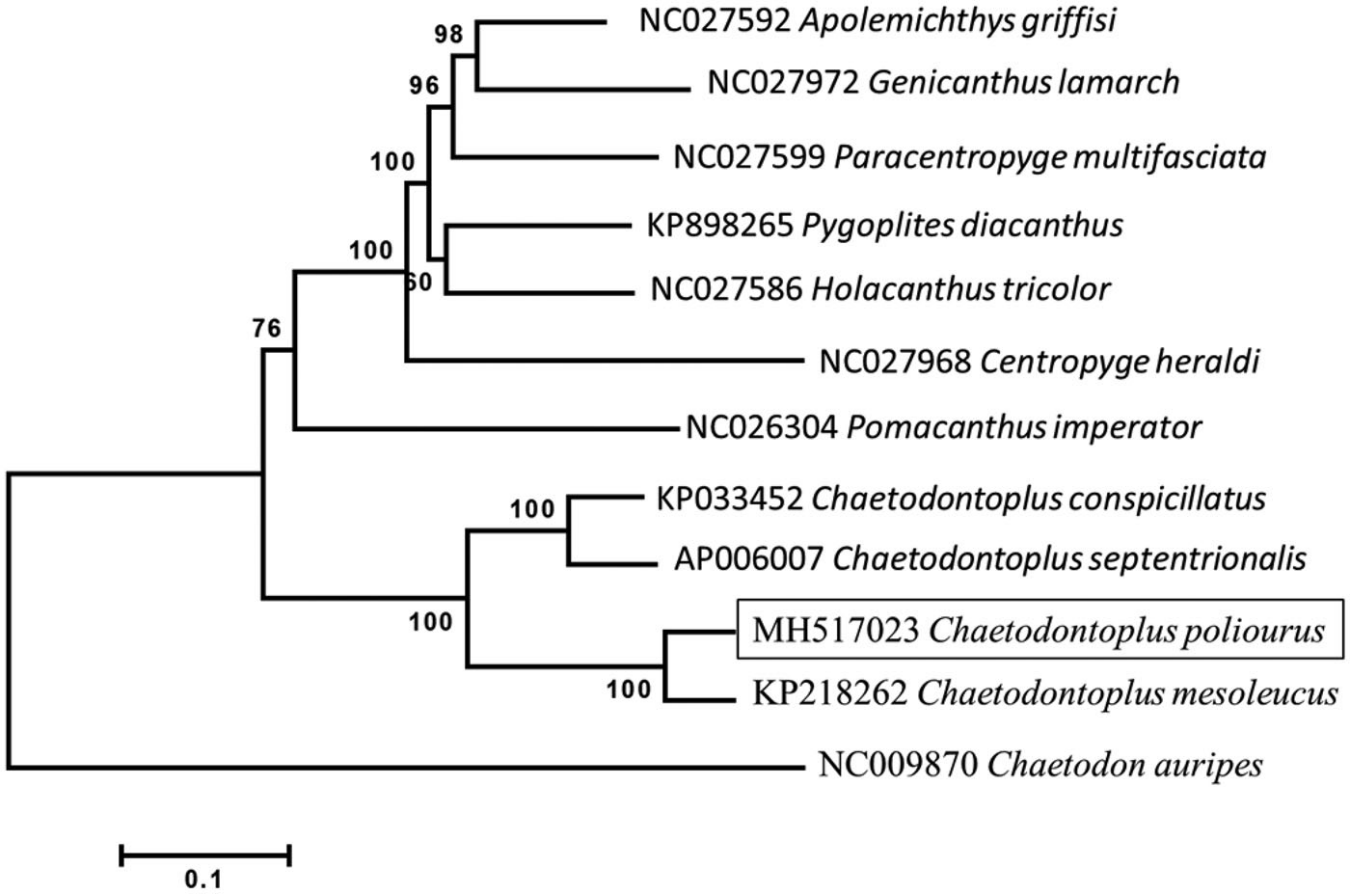
Maximum likelihood phylogenetic tree of Pomacanthidae with newly described species *Chaetodontoplus poliourus* (Square) under the GTR + G + I mutation model. The oriental Butterflyfish *Chaetodon auripes* was used as outgroup. The number in front of the species name is the NCBI accession number. The number at the root is the bootstraps value after 1000 replicates.

## Data Availability

The genome sequence data that support the findings of this study are openly available in GenBank of NCBI at (https://www.ncbi.nlm.nih.gov/) under the accession no. MH517023. The associated BioProject, SRA, and Bio-Sample numbers are PRJNA686515, SRR13276947-8, and SAMN17119470, respectively.
